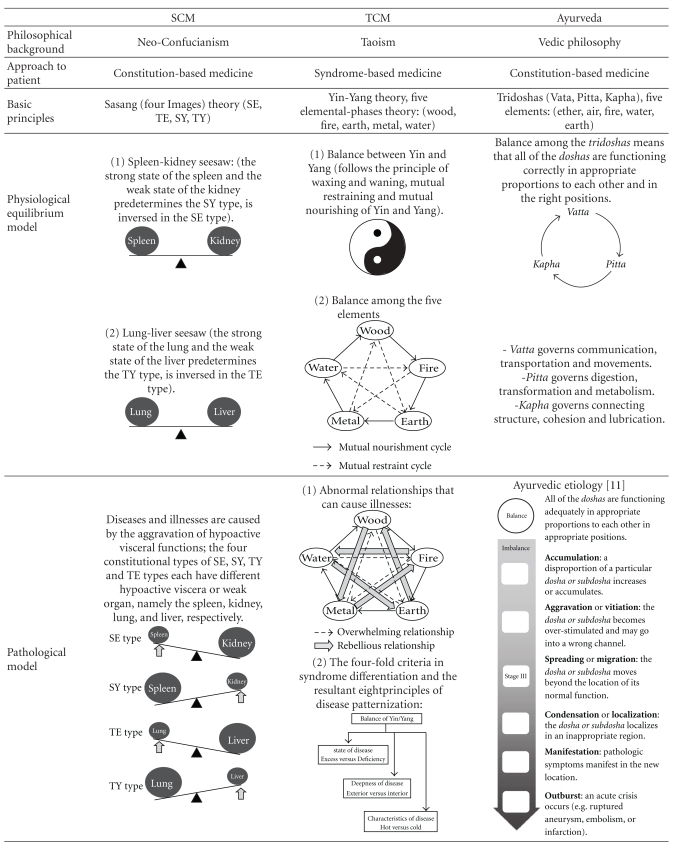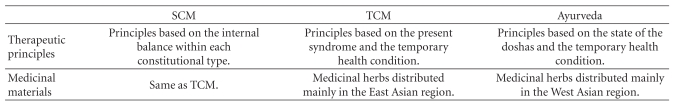# Comparison of Sasang Constitutional Medicine, Traditional Chinese Medicine and Ayurveda

**DOI:** 10.1093/ecam/neq052

**Published:** 2011-03-15

**Authors:** Jong Yeol Kim, Duong Duc Pham, Byung Hee Koh

**Affiliations:** ^1^Korea Institute of Oriental Medicine, 483 Exporo (461-24 Jeonmin-dong) Yuseong-gu, Daejeon 305-811, Republic of Korea; ^2^University of Science and Technology, 113 Gwahangno. Yuseong, Daejeon 305-333, Republic of Korea; ^3^National Hospital of Traditional Medicine of Vietnam, 29 Nguyen Binh Khiem St., Hai Ba Trung Dist., Hanoi, Vietnam; ^4^Kyunghee University, Department of Sasang Constitutional Medicine, Kyunghee University Medical Center, 1 Hoegi-dong, Dongdaemun-gu, Seoul 130-702, Republic of Korea

## Abstract

Sasang constitutional medicine (SCM), traditional Chinese medicine (TCM) and Ayurveda are three different forms of Asian traditional medicine. Although these traditions share a lot in common as holistic medicines, the different philosophical foundations found in each confer distinguishing attributes and unique qualities. SCM is based on a constitution-based approach, and is in this way relatively more similar to the Ayurvedic tradition than to the TCM, although many of the basic SCM theories were originally derived from TCM, a syndrome-based medicine. SCM and TCM use the same botanical materials that are distributed mainly in the East Asian region, but the basic principles of usage and the underlying rationale are completely different from each other. Meanwhile, the principles of the Ayurvedic use of botanical resources are very similar to those seen in SCM, but the medicinal herbs used in Ayurveda generally originate from the West Asian region which displays a different spectrum of flora.

## 1. Introduction

The two most prevalent forms of traditional medicine (TM) in Asia are the traditional Chinese medicine (TCM) and the traditional Indian medicine (represented by Ayurveda). Over the years, TCM and Ayurveda have evolved and spread around the world. The two medical traditions occupy an increasingly larger portion in the global market, presumably due to the rising interest not only among the consumers but also among medical practitioners [1]. The historical, cultural and social foundations of the Asian states were cultivated on top of the three main philosophical traditions, namely, the Vedic philosophy (giving rise to Ayurveda), Taoism (giving rise to TCM) and Confucianism. Ayurveda and the Vedic philosophy are predominant in the West Asian countries (India, Pakistan, Tibet, etc.), whereas TCM is practiced in the East Asian countries (China, Korea, Japan, Vietnam, etc.) [2]. Although it originated from TCM, traditional Korean medicine (TKM) eventually evolved into an independent medical tradition with distinctive qualities of its own. On a similar note, the Sasang constitutional medicine (SCM), first introduced by Jema Lee in 1894, is an indispensable part of TKM, which is generally based on the TCM theories [3]. Unlike TCM, SCM lays emphasis on a “patient-centered” approach in contrast to the “syndrome-centered” approach seen in TCM. SCM has won increasingly more popularity in Korea and overseas as more people began to recognize the effectiveness of SCM therapy and the advantages of its constitutionally individualized approach. Unlike conventional TM, the SCM is rooted in the neo-Confucianism philosophy and holds a constitution-based perspective [4]. Ayurveda, TCM and SCM share many aspects in common and yet have individual qualities that make each medical tradition unique and special. On the whole, SCM displays more similarity to Ayurveda than to TCM despite the geographical proximity between Korea and China.

This article portrays the basic picture of the three medical traditions of Ayurveda, TCM and SCM and sheds light on their similar and distinctive features.

## 2. Similarities between SCM, TCM and Ayurveda

SCM, TCM and Ayurveda share the basic holistic approach to healthcare in that an individual is assessed as a whole entity. According to these medical traditions, pathological conditions are the results of single or combined disturbances/imbalances on the physical, psychological, social and spiritual levels. Medical interventions therefore necessarily take into account the multifaceted and complex relationship between the spirit, mind and body, and the aim of therapy is not the elimination of the isolated disease or symptom but the treatment of the body as a whole [5].

TM diagnosis relies on subjective examination (observing, listening, inquiring and palpating) of the patient by the medical professional, and TM therapy includes a wide spectrum of therapeutic modalities such as herbal medication, acupunctural therapy and manual therapy.

SCM and TCM use medicinal materials from similar sources of medicinal plants available in the East Asian region, whereas Ayurveda utilizes the indigenous herbs of Western Asia. TM herbal remedies are generally a mixture of several medicinal herbs, and the synergistic effect of the ingredients produces the intended therapeutic results. The complex therapeutic effect rectifies the external disturbance to the body and restores the internal imbalance in the body [4, 6].

In this holistic approach, the patient is examined, assessed and treated as a whole, complex organism. TM therapy customizes the therapeutic methods applied to a specific pathology according to the individual patient's condition. For example, the same disease may be treated with different herbal formulae or therapeutic methods depending on several factors. Unlike other medical traditions that modulate their therapeutic protocol according to the particular pathologies, this approach can provide individualized and customized therapy to the patient [5].

Also, whereas TCM is primarily concerned about the symptoms or symptomatic manifestation, SCM and Ayurveda emphasize the enhancement of the patient's constitutional health condition.

## 3. Syndrome-Based Medicine and Constitution-Based Medicine

Individuals are born with different traits and characteristics. SCM and Ayurveda emphasize the importance of variation in the constitutional makeup among individuals. These two medical traditions are based on the recognition and acceptance of the inherent constitutional differences between individuals, a concept that is central in SCM and Ayurvedic therapeutics [3, 9]. In contrast, the pathological presentation of the patient at the time of examination is the foremost consideration in TCM, whereas the other factors (such as the progression of disease, family history and congenital conditions) are taken into consideration but only in a secondary capacity. Although TCM, SCM and Ayurveda share the qualities of holistic medicine in that they all treat an individual as a whole, they each start off from different viewpoints. TCM therapy begins with the evaluation and differentiation of syndrome (or the identification of disease patterns) [10], whereas the constitutional typing and determination of the constitutional proclivity are the first steps in SCM and Ayurveda therapy. Whereas the TCM therapy uses reducing and tonifying methods to redeem the external pathogenic factors such as blood stasis and qi deficiency, the therapeutic goal in SCM lies in the restoration and minimization of the imbalance in the quadrifocal organ scheme. In other words, although the therapeutic methods and materials may overlap, TCM and SCM use them for completely different reasons from completely different rationales.

Ayurveda assigns an individual into one of the seven main constitutional types, or *prakriti*, based on the inherent imbalance of the three energy forces, or *dosha*, that are each called *Vatta*, *Pitta* and *Kapha*. SCM is rooted in the quaternity central to the Sasang philosophy and classifies the constitutional makeup of an individual into one of the four constitutional types namely, the Taeyang type (TY), the Soyang type (SY), the Taeeum type (TE) and the Soeum type (SE) (Table 1). In SCM, the inherent proclivity in the constitutional imbalances exacerbates the weaknesses of the constitutional type, leading to specific patterns in susceptibility to particular pathologies. SCM therapy, therefore, is focused on minimizing these weaknesses in order to restore the internal balance [3, 9].

## 4. Similarities and Differences in Physiology and Pathology

In SCM, the physiological and pathological concepts are based on the quadrifocal scheme or quaternity (Sasang; 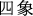) that varies from the bifocal scheme or dichotomy found in the Yin-Yang theory, the philosophical basis in TCM. As such, the internal organ structure in SCM can be summarized in a seesaw model; the seesaw model that can be seen in the SE and SY types is the spleen-kidney seesaw, where the spleen controls the intake of food and the kidney controls the discharge of waste products, and the seesaw model that can be seen in the TE and TY types is the lung-liver seesaw, where the lung controls the consumption of qi and fluid and the liver controls the storage of qi and fluid [4]. The SE type is characterized by a strong kidney system and a weak spleen system, whereas the SY type is characterized by a strong spleen system and a weak kidney system. The TE type is characterized by a strong liver system and a weak lung system, whereas the TY type is characterized by a strong lung system and a weak liver system (Table 2) [3, 4]. The concept of lung, liver, spleen and kidney in SCM was originally derived from the TCM theories but later evolved into a different, separate physiopathological concept. 

According to SCM, the requisite energy (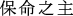), or the preservative energy related to the most hypoactive viscera or the weakness of each constitutional type, is considered to be the essential energy necessary to maintain homeostasis. The clearing Yin energy, the warming Yang energy, the dispersive energy and the accumulative energy are the requisite energies for the SE, SY, TE and TY types, respectively. Therefore, strengthening and preserving these requisite energies are the ultimate therapeutic goals in SCM [12].

The basic TCM pathology is also based on the Yin-Yang theory (as in SCM), but most of its physiological explanations, including the organ structure theory, are based on the five elemental phases theory. The five elemental phases are represented by wood, fire, earth, metal and water, and there exists a mutually assisting and controlling relationship (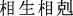) between them. The balance between these relationships is the therapeutic goal in TCM (Table 2).

The Ayurvedic physiology and pathology are based on the theory of the five elements suggested in the Vedic philosophy (ether, air, fire, water and earth) [13]. Although perhaps similar at first glance, this concept of the five elements in Ayurveda is completely different from that of the five elemental phases theory found in TCM. The two theories deal with similarly named but actually different elements; the five elements in the Ayurvedic theory are depicted to have a sequentially fortifying relationship only (Table 2), whereas the TCM elemental phases interact mutually in assisting and controlling relationships (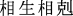).

## 5. Similarities and Differences in Treatment

The therapeutic modalities found in Asian TM traditions are generally based on botanical sources. The medicinal herbs used in SCM are similar to those used in TCM, but the basic principles of usage and the underlying rationale are completely different. In SCM, the constitutional type of the patient is the primary consideration in selecting the medicinal herbs and formulae for treatment. A particular medicinal herb is compatible with only one specific constitutional type and can therefore be used for that constitutional type only and be mixed with other herbs compatible with that constitutional type only. Use of a medicinal herb on an incompatible constitutional type can result in little effect or even induce adverse effects. For example, Radix Ginseng, an SE medicinal herb, and Radix Rehmanniae Glutinosae, an SY medicinal herb, should not be used in combination with each other. Also, a medicinal herb cannot be used across different constitutional types, but can be used for different symptomatologies or diseases within that constitutional type [14, 15].

In contrast, TCM medicinal herbs are classified according to the therapeutic effects of the herb itself, namely, dispersive quality, Yin tonifying quality and so forth. Consequently, a particular medicinal herb can be applied to any patient afflicted with the same disease or pathology regardless of the individual's constitutional type. For instance, Radix Ginseng is sometimes used in combination with Radix Rehmanniae Glutinosae in some TCM formulae [16].

Ayurvedic and SCM therapeutics are based on constitutional approach, and the medicinal herbs are selected or excluded according to their compatibility or incompatibility to the constitutional makeup of a given individual. Ayurvedic medicinal herbs are distinguished by their effects on the three *doshas*, whereas SCM medicinal herbs are categorized according to their effects on the different constitutional types. For instance, Cortex Cinnamomi, a commonly used medicinal herb, is described in the Ayurvedic practice as being able to repress *Vitta* and *Kapha* while enhancing *Pitta*, whereas in SCM it is suggested to be compatible with the SE type and incompatible with the SY type. On a slightly different note, the actual specimen of medicinal herbs used in Ayurveda and SCM are likely to be different from each other due to the differences in the regional flora [4, 13].

## 6. Conclusion

The three forms of TM, Ayurveda, TCM and SCM, are unique medical traditions originating in the Asian region. This brief comparison shows that SCM shares much of the same basic theories with TCM (e.g., the Yin-Yang theory and the medicinal herbology) but is still a separate, independent medical tradition that has developed a different theoretic basis and unique fundamental concepts that are rooted in the constitution-based approach. In this, SCM can be said to be more different from TCM and relatively more similar to the Ayurvedic tradition.

## Figures and Tables

**Table 1:** General characteristics of the constitutional types in Ayurveda and SCM.

Ayurvedic body type (prakriti) [7]			Distinctive factors		Sasang constitutional type [8]			
*Vatta *	*Pitta *	*Kapha*			TY	SY	TE	SE
			Physical					
Lean	Medium	Well built	Physique		Developed in the nape area,slender waist	Developed in the chest area, small hip	Developed in the waist area, frail nape	Developed in the hip area, weak chest
Dry, dark, with superficial veins and tendons	Bright skin with moles	Oily, whitish	Skin texture		Slippery	Slippery, thin	Thick, stiff, rough	Soft, feeble, smooth
Rough, scanty	Soft, brown, grays early or bald	Thick, black, lustrous	Hair*	Facial features**	Big and round face, wide forehead, large ears	Small, round and bulging face, twinkling eyes, high and pointy nose, thin and small lips	Wide jaw, wide distance between eyebrows, big eyes, thick ears, thick lips	Ellipsoid face, less predominant forehead, low upper eyelids, big ear lobes, small nose
Dry, thin, small	Pink, soft	Thick, oily, large	Nails*	Voice**	Powerful, loud, clear ringing voice	High pitched, plain, talkative	Low pitched, powerful, vague	Weak, powerless, light and clear

			Physiological					
Inconsistent	High	Low	Appetite		Moderate	Good	Good, overeating	Poor, dyspeptic
Demands small quantity, eats fast	Demands large quantity, eats frequently	Eats less and slowly	Eating habit		Moderate	Eats fast	Requires large quantity	Requires small quantity, eats slowly
Dry, hard stool, constipation tendency	Loose stool, irritable tendency	Solid, oily stool	Bowel movement		Constipated	Constipated	No specific tendency	Soft stool, frequent
Inconsistent	High	Low	Digestion rate		Good	Good	Good	Low
Fast and rapid	Moderate	Slow	Movements		Quick	Moderate	Slow	Slow
N°	N°	N°	Sweating**		Moderate	Moderate	Profuse	Mild
N°	N°	N°	Sign of health**		Smooth urination	Easy defecation	Brisk perspiration	Comfortable digestion

			Psychological					
Inconsistent	Intelligent	Slow, dull	Mental nature		Progressive	Unstable, hot tempered	Gentle, materialistic	Meticulous, gentle, pessimistic

*Factors of concern only in the Ayurvedic determination of the constitution. **Factors of concern only in the SCM determination of the constitution. N°, no information.

**Table 2:** A comparison between SCM, TCM and Ayurveda.